# Epstein-Barr virus infection and oral squamous cell carcinoma risk: A meta-analysis

**DOI:** 10.1371/journal.pone.0186860

**Published:** 2017-10-24

**Authors:** Yangyang She, Xiaolin Nong, Min Zhang, Menglin Wang

**Affiliations:** 1 Department of Oral and Maxillofacial Surgery, College and Hospital of Stomatology, Guangxi Medical University, Nanning, Guangxi, China; 2 Department of Prosthodontics, College and Hospital of Stomatology, Peking University, Beijing, China; 3 Department of Otorhinolaryngology and Head and Neck Surgery, The First Affiliated Hospital of Guangxi Medical University, Nanning, Guangxi, China; Southern Illinois University School of Medicine, UNITED STATES

## Abstract

**Background:**

The evidence for association between Epstein-Barr virus (EBV) infection and risk of oral squamous cell carcinoma (OSCC) is inconsistent in the literature. Therefore, this meta-analysis was conducted to clarify this association.

**Methods:**

A literature search was conducted in electronic databases for English- and Chinese-language publications until March 31, 2017 to include eligible case-control studies. The pooled *odds ratio* (*OR*) and 95% *confidence interval* (95% *CI*) were estimated to determine the association between EBV infection and OSCC risk using a fixed- or random-effects model based on heterogeneity. Publication bias was assessed using funnel plot analysis.

**Results:**

A total of 13 case-control studies with 686 OSCC patients and 433 controls were included based on predetermined inclusion and exclusion criteria. The pooled *OR* with 95% *CI* between EBV infection and OSCC risk was 5.03 (1.80–14.01) with significant heterogeneity observed (*I*^*2*^ = 87%). The subgroup analysis indicates that the year of publication, study location, economic level, sample size, tissue type, detection method and marker, control type, and language might explain potential sources of heterogeneity. Publication bias was not observed, and sensitivity analysis showed stable results.

**Conclusions:**

The results of the current meta-analysis suggest that EBV infection is statistically associated with increased risk of OSCC. However, additional high-quality studies with larger sample sizes are needed to further confirm the relationship between EBV and OSCC.

## Introduction

Oral squamous cell carcinoma (OSCC) is the most common subset (90%) of oral cancer with a global incidence of 275,000 cases annually [1], the sixth leading malignancy worldwide [2]. It results from the outgrowth of the mucosal epithelium. Local recurrence and regional and distant metastases can occur even decades after surgery, radiation, and chemotherapy, making OSCC life-threatening [3]. The 5-year survival rate of late-stage OSCC is only 20% [1]. The high disease burden and low survival rate highlight the need to better understand the etiology of OSCC. Established risk factors for OSCC involve long-term betel quid chewing, tobacco smoking, and alcohol drinking [4–6]. In addition, numerous other possible risk factors have also been proposed. Recently, it has been demonstrated that individuals with Epstein-Barr virus (EBV) infection might be at increased risk for OSCC.

EBV is an oncogenic human herpes virus that contains double-stranded DNA, known as the first human tumor virus [7,8]. It appears even in asymptomatic individuals, persisting for lifelong latent infection [7]. EBV has been well proposed as a causative agent for several types of epithelial cell malignancies, such as nasopharyngeal carcinoma (NPC) [9]. Furthermore, evidence has shown that EBV is involved in B-lymphocytic cell malignancies, such as Burkitt’s lymphoma and Hodgkin lymphoma [7,10].

The strength and consistency of EBV DNA present in OSCC indicate a potentially important role of EBV infection on OSCC pathogenesis. However, controversial results have been reported [11–13]. Therefore, a meta-analysis was conducted to assess the association between EBV infection and OSCC risk.

## Methods and materials

### Search strategy

The current meta-analysis was conducted based on the Meta-Analysis of Observational Studies in Epidemiology guidelines [14]. Pubmed, Web of Science, Cochrane, and Embase databases for English-language publications and China National Knowledge Infrastructure (CNKI), Wanfang Data, Chinese Scientific Journals Fulltext Database (CQVIP), and China Biology Medicine disc (CBM disc) for Chinese-language publications were searched until March 31, 2017. Keywords used for the Pubmed search are as follows: Search ((((“Epstein-Barr Virus”[Title/Abstract]) or “EBV”[Title/Abstract]) or “human herpesvirus 4”[Title/Abstract]) or “HHV 4”[Title/Abstract]) and ((((((((“oral squamous cell carcinoma”[Title/Abstract]) or “OSCC”[Title/Abstract]) or “oral carcinoma”[Title/Abstract]) or “oral cancer”[Title/Abstract]) or “tongue cancer”[Title/Abstract]) or “buccal cancer”[Title/Abstract]) or “oral lesions”[Title/Abstract]) or (“head and neck cancer”[Title/Abstract])). Similar search was conducted in the Web of Science, Cochrane, and Embase databases. The references lists of the retrieved articles and previous systematic reviews were also reviewed to identify potential eligible studies.

### Study selection

The titles and abstracts of all relevant studies were independently examined by two authors (YYS and MZ) to assess eligibility. Studies were included based on the following criteria: (1) evaluated the association between EBV infection and patients with OSCC through the expression of EBV level (DNA, RNA, or protein) in tissue samples; (2) confirmed histopathological diagnosis of OSCC cases; (3) reported original data; (4) used a case-control study design; (5) employed fresh, frozen, or paraffin-embedded (PE) storage methods; (6) used polymerase chain reaction (PCR), reverse transcription PCR (RT-PCR), real-time quantitative PCR (qPCR), *in situ* hybridization (ISH), and immunohistochemistry (IHC); and (7) full text available in English or Chinese. Case series, reports, animal models, in vitro studies, reviews, editorials, conference abstracts, and letters without sufficient data were excluded. If multiple publications reported results based on the same study, the most recent article or the article with bigger sample size was included.

### Data extraction

The data extraction was performed by two authors (YYS and MZ) independently using a pre-designed data extraction form based on the guidelines for meta-analysis [14]. Discrepancies were adjudicated by discussing or consulting with a third author (XLN). The following information was extracted: last name of the first author, year of publication, study location, number of OSCC cases, number of controls, sample size, tissue type, detection method and marker, and control type.

### Quality assessment

The Newcastle-Ottawa Scale (NOS) was used to evaluate the methodological quality of the included studies [15], where five stars indicate moderate to high quality.

### Statistical methods

The numbers of OSCC cases and controls were used to calculate the pooled *odds ratios* (*ORs*) and the corresponding 95% *confidence intervals* (*CIs*) based on weighted pooled measures. Forest plots were generated to visually evaluate the study-specific and pooled effects. Heterogeneity across studies was evaluated using the Cochran’s *Q* test with a significant level of *P* <0.10 or the *I*^*2*^ statistic >50% [16,17]. The random-effects model was used if *P* <0.10 or *I*^*2*^ >50%. Otherwise, the fixed-effects model was used [18]. Sensitivity analysis was performed by omitting one study at a time to examine the influence of an individual estimate on the pooled estimates. Subgroup analyses were also conducted based on key study characteristics (the year of publication, study location, economic level, sample size, tissue type, detection method and marker, control type, and language) which might be the potential sources of heterogeneity. Meta-regression was applied to evaluate the effects of the aforementioned variables on the association between EBV infection and OSCC risk. The cumulative meta-analysis was performed based on the year of publication to observe the temporal trend of the cumulative estimate with increasing sample sizes [19]. Begg’s and Egger’s tests were performed to examine the potential publication bias [20,21]. Funnel plot was generated to examine potential publication bias through the visual inspection of asymmetry [22]. All statistical analyses were performed with Stata Version 11.0 (Stata Corp, College Station, Texas). All reported *P* values were two-sided, with *P* <0.05 considered statistically significant, except where otherwise specified.

## Results

### Literature selection

**Fig 1:**
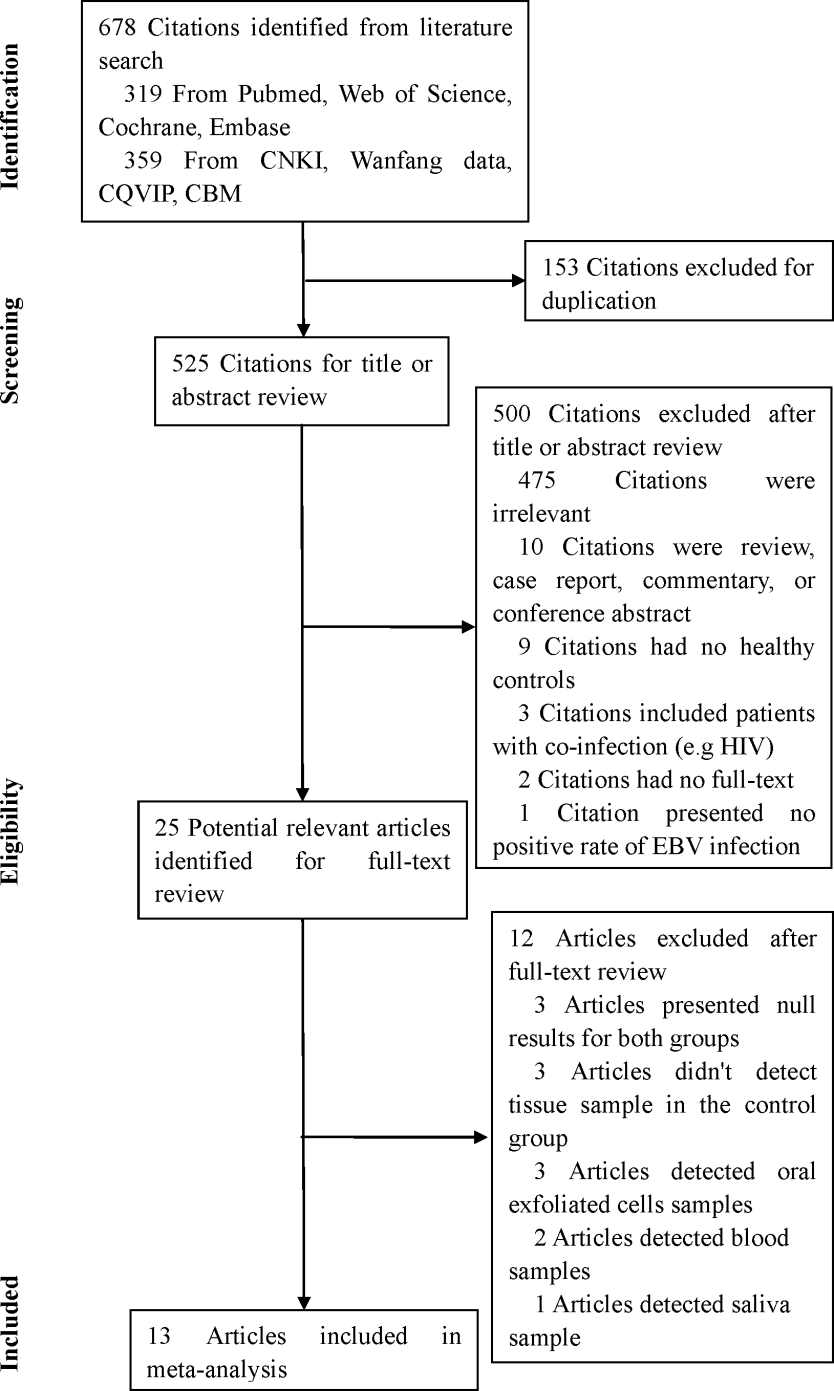
Flow diagram of the study selection process and results of the literature search.

A total of 678 citations were retrieved from Pubmed, Web of Science, Cochrane, Embase, CNKI, Wanfang data, CQVIP, and CBM. After excluding 153 citations due to duplication, 525 unique citations were considered further. Of these, 500 citations were sequentially excluded after the first screening based on the abstracts or titles (Fig 1). The full texts of the remaining 25 articles were reviewed. After excluding 12 studies, the remaining 13 studies [11–13,23–32] were finally included in this meta-analysis.

### Study characteristics

Table 1 shows the characteristics of the 13 included studies. The included studies were published between 1995 and 2016. The total number of participants in case and control groups was 686 and 433, respectively. Two studies were conducted in China, two in Japan, two in South Africa, and the other seven were conducted in India, Egypt, Sweden, the Netherlands, Spain, Hungary, and USA. All 13 studies used tissue samples for EBV detection; nine of these used PE tissues and the other four used fresh/frozen tissues. Eight and two studies applied PCR and Nested PCR, respectively, to detect EBV DNA, and the remaining three studies applied RT-qPCR, ISH, and IHC to detect EBV RNA (EBV-encoded small non-polyadenylated RNA 1, i.e., EBER1), EBV DNA (Bam HIW fragment), and EBV protein, respectively. The included studies selected normal oral tissues as controls, including two randomly selected normal oral tissues, two age-matched normal oral tissues, one paracancerous normal oral tissues, one contralateral normal oral tissues, and seven used normal oral tissues. The methodological quality of the included studies were moderate or high with at least five scores, except for one study with only four scores based on the NOS.

**Table 1:** Summary of studies included in the meta-analysis.

Study [Reference]	Year	Study location	Number of case		Number of control		Sample size	Detection method ^a^	Detection marker ^b^	Tissue type ^c^	Control type	NOS Score ^d^
			EBV(+)	EBV(-)	EBV(+)	EBV(-)						
Rensburg, et al[11]	1995	South Africa	13	35	16	22	86	PCR	EBV DNA(BamHIW)	PE tissue	Normal oral tissue	7
Heerden, et al[12]	1995	South Africa	22	68	11	19	120	PCR	EBV DNA(BamHIW)	PE tissue	Normal oral tissue	5
Ctuz, et al[13]	1997	The Netherlands	36	0	1	11	48	PCR	EBV DNA(BamHIW)	frozen tissue	Normal oral tissue	6
Ding, et al[23]	1997	China	20	40	4	36	100	PCR	EBV DNA	PE tissue	Normal oral tissue (random)	6
Chen, et al[24]	1998	China	18	3	7	14	42	PCR	EBV DNA	fresh tissue	Paracancerous normal tissue	4
D'Costa, et al[25]	1998	India	25	78	3	73	179	PCR	EBV DNA	frozen tissue	Contralateral normal tissue	6
Shimakage,et al[26]	2002	Japan	30	6	0	3	39	ISH	EBV DNA(BamHIW)	PE tissue	Normal oral tissue	7
Sand, et al[27]	2002	Sweden	11	18	5	62	96	Nested PCR	EBV DNA	PE tissue	Normal oral tissue (age-matched)	7
Shamaa, et al[28]	2008	Egypt	18	4	0	20	42	IHC	EBV protein	PE tissue	Normal oral tissue	7
Bagan, et al[29]	2008	Spain	2	3	0	5	10	Nested PCR	EBV DNA	frozen tissue	Normal oral tissue(random)	5
Kis, et al[30]	2009	Hungary	48	17	13	55	133	PCR	EBV DNA(BamHIW)	PE tissue	Normal oral tissue (age-matched)	8
Jiang, et al[31]	2012	USA	11	10	7	16	44	RT-qPCR	EBV RNA(EBER1)	PE tissue	Normal oral tissue	5
Kikuchi, et al[32]	2016	Japan	78	72	25	5	180	PCR	EBV DNA(EBNA2)	PE tissue	Normal oral tissue	5

^a^ PCR: polymerase chain reaction, ISH: in situ hybridization, IHC: immunohistochemical, RT-qPCR: reverse transcription and quantitative real-time polymerase chain reaction

^b^ EBER1: EBV-encoded small non-polyadenylated RNA 1, EBNA2: EBV-determined nuclear antigens 2

^c^ PE: paraffin-embedded

^d^ NOS: Newcastle-Ottawa Scale

### The association between EBV infection and OSCC

**Fig 2:**
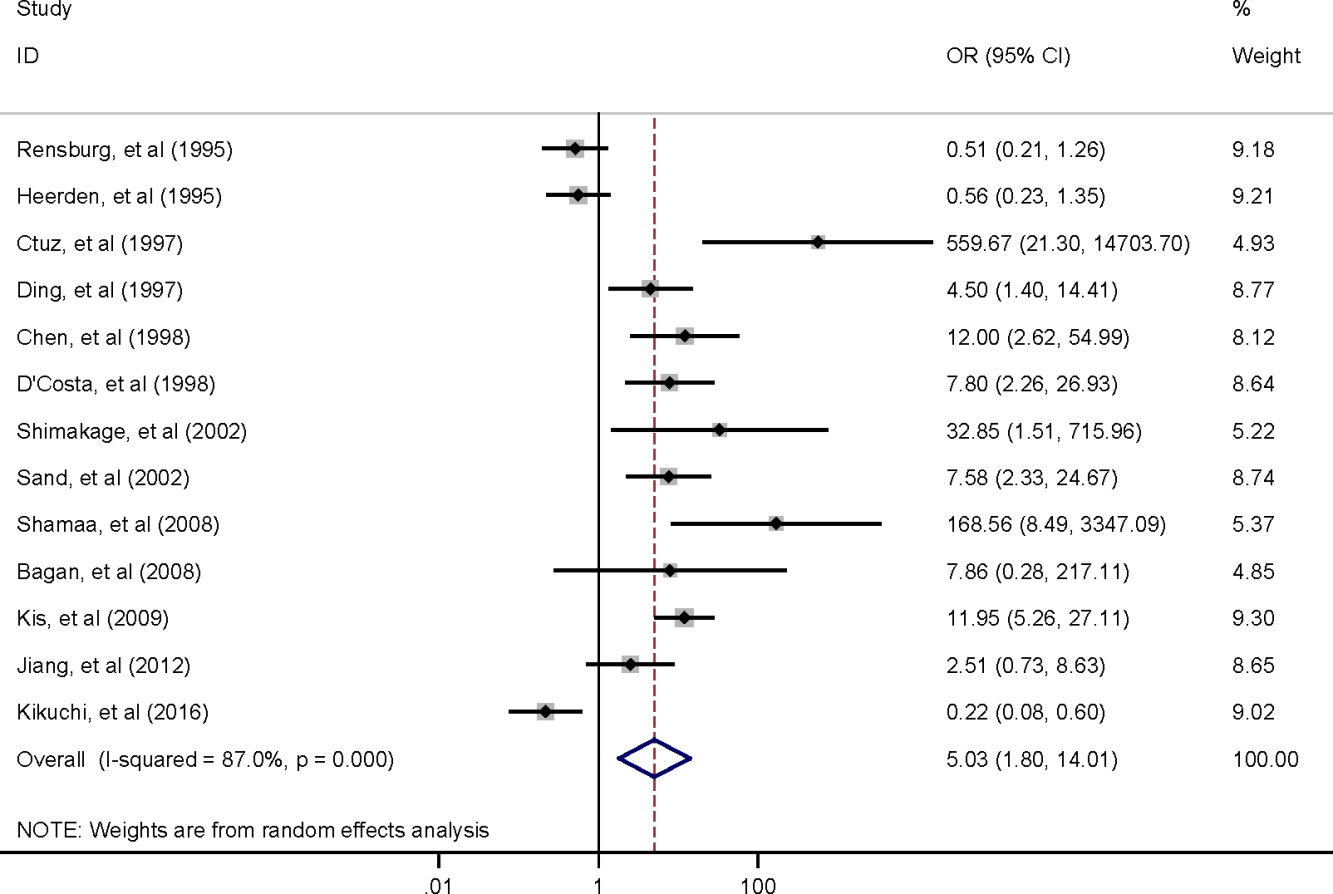
Forest plot of the association between EBV infection and OSCC risk.

**Fig 3:**
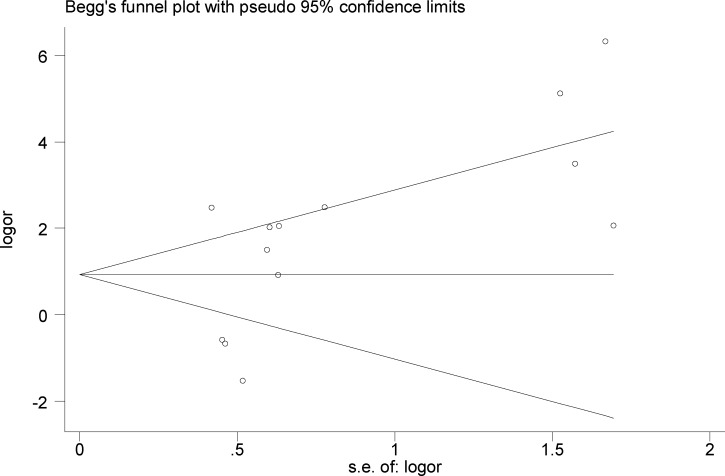
Sensitivity analyses by omitting individual study.

Among the 13 studies, the pooled association between EBV infection and OSCC risk was *OR* 5.03 (95% *CI*, 1.80–14.01) (Fig 2). Sensitivity analysis revealed stable results. The estimates did not vary materially ranging from 3.87 (95% *CI*, 1.42–10.53) to 6.53 (95% *CI*, 2.45–17.40) (Fig 3).

### Heterogeneity and subgroup analysis

To explore the heterogeneity of the study, subgroup analyses and meta-regression on a number of key study characteristics were performed (Table 2). EBV infection and OSCC risk were consistently and positively associated in all subgroups, although not all estimates were statistically significant. The pooled *OR*s (95% *CI*s) for the association of EBV infection and OSCC from different study locations were 4.17 (0.69–25.21), 9.69 (3.16–29.72), and 1.96 (0.24–15.91) in Asia, Europe and USA, and Africa, respectively; 3.83 (0.94–15.53) and 6.92 (1.35–35.40) for developing and developed countries, respectively; 2.17 (0.44–10.80) and 10.47 (2.39–45.79) for sample sizes <100 and ≥100, respectively; 4.25 (0.99–18.29) and 6.17 (1.27–29.95) for studies published before and on or after 2000, respectively; 2.39 (0.78–7.33) and 31.27 (5.79–169.01) for PE and fresh/frozen tissues, respectively; 3.37 (0.90–12.65) and 7.62 (2.50–23.22) for studies applying PCR and Nested PCR, respectively; 4.36 (1.42–13.40) and 16.76 (0.22–1276.16) for studies detecting EBV DNA, and EBV RNA and protein, respectively; 4.78 (1.60–14.33), 10.37 (5.30–20.28), and 4.37 (1.16–16.97) for studies randomly selected, age-matched, and neither random nor age-matched control samples, respectively; and 6.29 (2.49–15.89), and 4.84 (1.48–15.88) for Chinese- and English-language publications, respectively. The meta-regression analysis revealed that the selected study characteristics did not significantly influence the results (*P* >0.05), except in the type of tissues (*P* = 0.03), and only partially explained the source of heterogeneity.

**Table 2:** Subgroup analysis of association between the EBV infection and OSCC risk.

Subgroup analysis	No. of Studies	Pooled *ORs* *(95% CI)* ^*f*^	*P* value	*I*^*2*^ (%)	*P* value	
					Heterogeneity	Meta-regression
**Overall**	13	**5.03(1.80–14.01)**	**0.002**	87	<0.001	**-**
**Study location**						
Asia	5	4.17(0.69–25.21)	0.120	88	<0.001	0.244
Africa	3	1.96(0.24–15.91)	0.530	88	<0.001	
Europe and USA	5	**9.69(3.16–29.72)**	**<0.001**	63	0.028	
**Economic level**						
Developing countries	6	3.83(0.94–15.53)	0.060	87	<0.001	0.678
Developed countries	7	**6.92(1.35–35.40)**	**0.020**	88	<0.001	
**Number of patients**						
<100	5	2.17(0.44–10.80)	0.342	92	<0.001	0.191
≥100	8	**10.47(2.39–45.79)**	**0.002**	83	<0.001	
**Publication year**						
Before 2000	6	4.25(0.99–18.29)	0.052	88	<0.001	0.801
2000 and After	7	**6.17(1.27–29.95)**	**0.024**	88	<0.001	
**Tissue type**						
PE ^a^	9	2.39(0.78–7.33)	0.128	88	<0.001	**0.033**
Frozen/Fresh	4	**31.27(5.79–169.01)**	**<0.001**	64	0.038	
**Detection method** ^b^						
PCR	8	3.37(0.90–12.65)	0.072	91	<0.001	0.507
Nested PCR	2	**7.62(2.50–23.22)**	**<0.001**	0	0.984	
RT-qPCR	1	-		-	-	
ISH	1	**-**		-	-	
IHC	1	**-**		-	-	
**Detection index**						
EBV DNA ^c^	11	**4.36(1.42–13.40)**	**0.010**	88	<0.001	0.533
EBV RNA and protein ^d^	2	16.76(0.22–1276.16)	0.202	86	0.007	
**Control type**						
Randomly selected	2	**4.78 (1.60–14.33)**	**0.005**	0	0.756	0.653
Age-matched	2	**10.37 (5.30–20.28)**	**<0.001**	0	0.534	
Other ^e^	9	**4.44(1.16–16.97)**	**0.029**	88	<0.001	
**Language**						
Chinese	2	**6.29(2.49–15.89)**	**<0.001**	1	0.315	0.826
English	11	**4.84(1.48–15.88)**	**0.009**	89	<0.001	

^a^ PE: paraffin-embedded

^b^ PCR: polymerase chain reaction, ISH: in situ hybridization, IHC: immunohistochemical, RT-qPCR: reverse transcription and quantitative real-time polymerase chain reaction

^c^ EBV DNA: Bam HIW, and EBV-determined nuclear antigens 2 (EBNA2)

^d^ EBV RNA: EBV-encoded small non-polyadenylated RNA 1 (EBER1)

^e^ other normal oral tissues neither randomly selected nor age-matched

^f^ Use fixed-effects model if *I*^*2*^ <50% or *P* value for heterogeneity >0.10

### Cumulative meta-analysis

**Fig 4:**
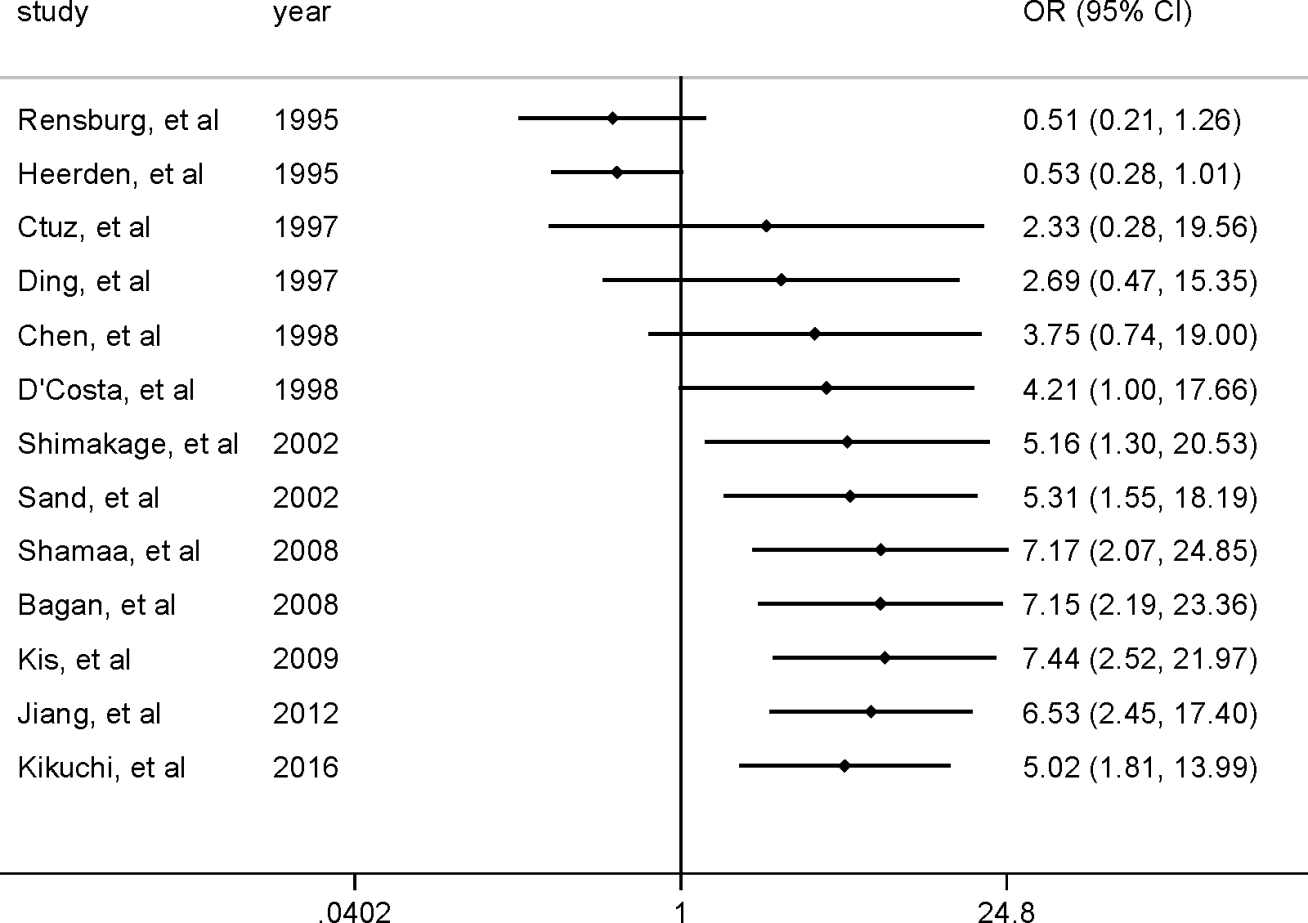
Cumulative meta-analysis for evaluating the temporal trend in the association between EBV infection and OSCC risk.

A cumulative meta-analysis was conducted to evaluate the temporal trend of the pooled results using random-effects model (Fig 4). Most cumulative results revealed a positive association between EBV infection and OSCC. The cumulative evidence showed a consistent positive association between EBV infection and OSCC from 1997 to 2016 with 2.33 (95% *CI*, 0.28–19.56) and 5.02 (95% *CI*, 1.81–13.99) cumulative estimates; however, the association between EBV infection and OSCC risk was relative weaker in the earlier than the more recent studies with a higher cumulative estimate and narrower 95% *CI*.

### Publication bias

**Fig 5:**
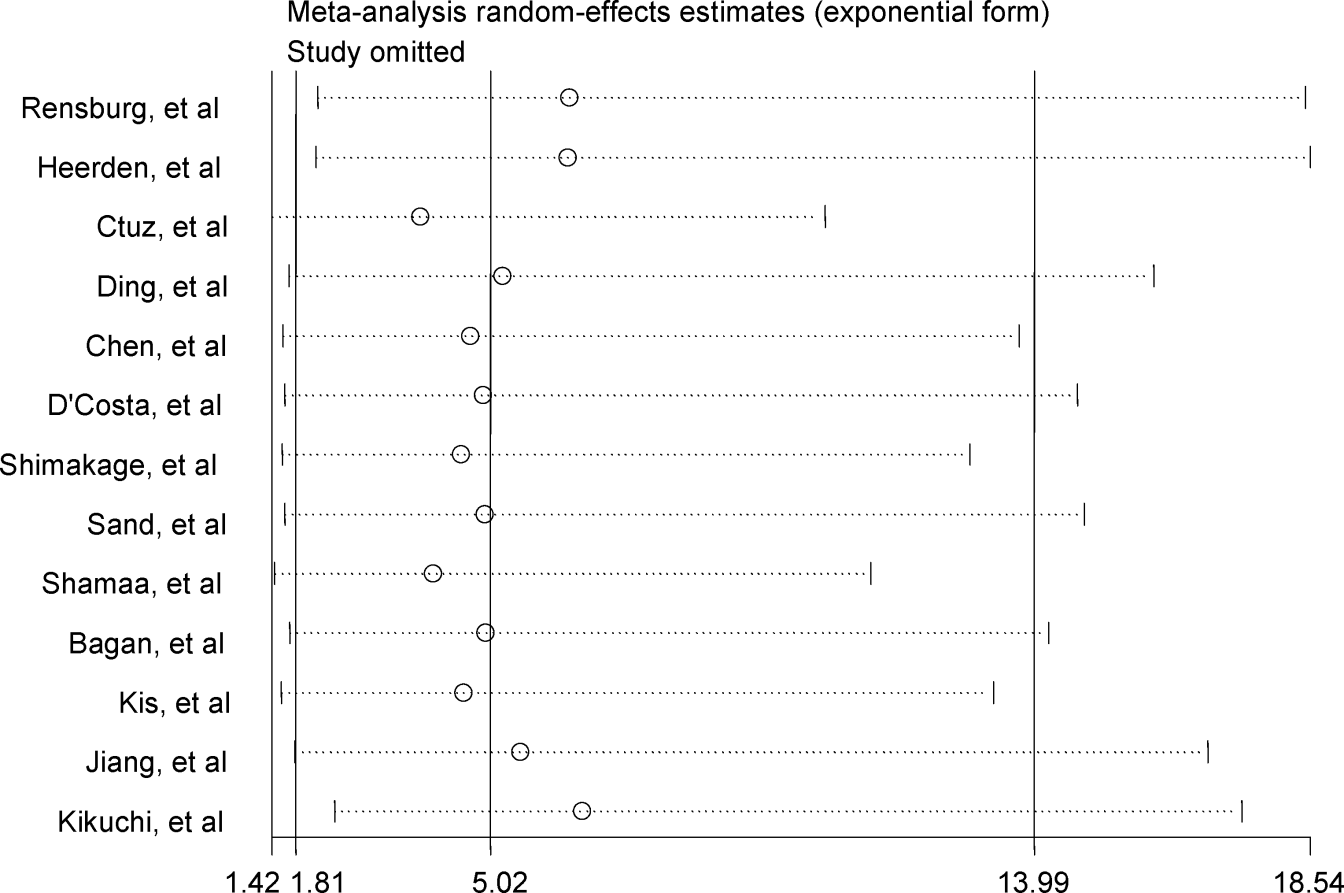
Funnel plot for publication bias regarding the association between EBV infection and OSCC risk.

No publication bias was observed (Begg’s test, z = 1.22, and *P* = 0.222; Egger’s test, *intercept* = 3.45, *t* = 1.98, and *P* = 0.073). Visual inspection of the Begg's funnel plot revealed a nearly symmetrical distribution, confirming the absence of publication bias (Fig 5).

## Discussion

The results of the 13 studies included in this meta-analysis suggest a positive association between EBV infection and OSCC risk, which was also shown in the results of the subgroup analyses, although not all of them were statistically significant. No publication bias was detected. In addition, the results were not driven by any single study based on the findings in the sensitivity analysis. Based on the results, EBV infection might be, therefore, associated with an increased risk of OSCC.

Viral infections were mediators of malignant proliferation in the head and neck squamous carcinoma [33]. An EBV-associated malignancy is associated with viral proteins that regulate the proliferation, immune response, and cell apoptosis [34]. EBERs are small noncoding RNAs and serve as active EBV infection markers. Latent membrane proteins (LMPs) aid in activating the signaling pathways connected to EBV persistence, whereas EBV-determined nuclear antigens (EBNAs) regulate gene expression. The EBV oncoprotein LMP-1 constitutively activates nuclear factor-kappa B (NFκB), which play a pivotal role in EBV-immortalized B-cells survival. Regarding to the EBV-encoded proteins, BHRF1 protein presents 25% sequence homology with the bcl-2 proto-oncogene and protects cells from apoptosis. LMP-1 and EBNA-5-protein inhibit p53-mediated apoptosis [33,34]. NPC has been associated with EBERs, EBNA1, LMP-1, LMP-2, and BARF0. The products of these genes affect the cell immortalization and viral genome replication [7]. However, whether EBV affects the carcinogenesis of the oral mucosal tissue remains uncertain. Positive reactivity of the EBV products might show similar contribution to OSCC cases.

The association between EBV infection and OSCC risk has been inconclusive in the available literature. The controversial results might be partly due to the differences in the methodologies or techniques applied to detect EBV in OSCC samples. Techniques used in detecting EBV, such as PCR, Nested PCR, RT-qPCR, IHC, and ISH, vary. Correspondingly, two general targets, namely, the viral products (encoded RNAs and proteins) and viral genome DNA, were used to detect EBV [26,28,29,31,32]. The sensitivity and specificity varied depending on the techniques, leading to different associations between EBV infection and OSCC [35]. Furthermore, several methods should be performed in a single experiment because of frequent weak expression of the transforming gene [26].

The results of this study indicated that EBV DNA, mRNAs, and EBV proteins were expressed in the majority of OSCC cells [26,28]. Two EBV DNA regions (Bam H1W and EBNA2) were detected in OSCC tissues (Table 1), showing various abilities in detecting EBV. Bam H1W was chosen in five studies that included 426 cases. A leader sequence contained using the Bam HIW fragment [36], which is supposed to be an oncogene, makes it a good biomarker for EBV. Kikuchi et al. [32] reported that the positive rate of EBNA2 in OSCC tissue was 50.2%, whereas the LMP-1 expression was 10.7% in the same sample. This analysis underlines the importance of the PCR marker on estimating the extent of the relationship between EBV infection and OSCC.

Based on our results, the fresh/frozen tissues showed a slightly higher EBV positivity rate (49.09%) than that of PE tissues (48.18%). The results of meta-regression indicate that different methods of sample storage might contribute to heterogeneity of the results (*P* = 0.03). However, the use of PE tissues promotes easier and simpler DNA detection. Indeed, according to a previous study, Deacon et al. [37] reported that PE tissues had lower detection rate compared with that of the fresh tissues.

Subgroup analyses based on the geographic region indicates a significant positive association between EBV infection and OSCC risk in Europe and USA, which is probably related to heredity and lifestyle. Different socioeconomic statuses might be associated with different EBV prevalence, leading to varied risk of OSCC between developed and developing countries [38].

The pooled association between EBV infection and OSCC risk differed by the year of publication in the studies published before 2000 and on or after 2000. The pooled estimate was greater among the studies published more recently than the earlier ones, as revealed in the cumulative meta-analysis. The pooled risks of OSCC with EBV infection among studies with larger sample sizes presented a statistically significant and higher estimate than those with smaller sample sizes, indicating that larger sample sizes could enable us to find smaller statistical difference [39].

Heterogeneity is a common problem in the meta-analyses and would weaken the validity and reliability of the results. Actually, EBV expression highly depends upon the sample type, probably due to the fact that >90% of adults are EBV seropositive and EBV is also often found in the saliva of asymptomatic patients. One possibility, for example, could be that the origin of viral genomes may be from the oropharynx and then appear in the saliva [7,40]. Therefore, our pre-specified inclusion criteria excluded those studies using peripheral blood, saliva, and oral exfoliated cell samples, we only chose studies detecting the pathological tissue samples to minimize heterogeneity. Studies evaluating the association between other factors and OSCC were excluded because this meta-analysis was mainly designed to assess the relationship between EBV infection and risk of OSCC. Immunosuppression, for example, caused by co-infection with HIV, may be an alternative factor that increases the risk of EBV to infect squamous cells [41,42]. There is an assumption that viral DNA only acts as a passenger in the OSCC cells or the OSCC cells are susceptible targets and easily get infected, as reported by Horiuchi et al. [43]. However, several other factors may result in a potential bias due to the techniques and assessment of expression, such as the inappropriate study design (neither selecting random nor age-matched sample), differences in techniques and methodologies for detection, interlaboratory variability when using the same test methods, histological classification of tumor tissues, and inaccurate definition of the normal tissues [44]. Further, results of the subgroup analyses and meta-regression reveal that the year of publication, study location, economic level, sample size, tissue type, detection method and marker, control type, and language might be the sources of heterogeneity, but heterogeneity was only partly explained.

To the best of our knowledge, this is the first meta-analysis investigating the relationships between EBV infection and OSCC risk. Nevertheless, a few limitations should be noted. First, high heterogeneity was shown in overall and subgroup analyses, which might add some uncertainty about the magnitude of the conclusion [45,46], and should be taken into consideration when interpreted the results. Second, all the included studies were published in English or Chinese, possibly leading to a language bias, although some previous studies suggested that it did not appear to influence the results [47,48]. Third, there is a concern regarding the potential risk bias. All included studies were retrospective studies, and the integrity of original data and potential recall bias might affect the results [45]. Fourth, the included studies were small-scale case control studies with small sample size, which might lower the precision of the results. Fifth, some studies failed to provide more details regarding the collection of control specimens. Only two studies specified that the control samples were randomly selected, and the other two studies selected age-matched control samples to reduce the selection bias. Finally, residual confounding may be likely. Some studies failed to control confounders such as gender, age, marital status [49], smoking, alcohol drinking, socioeconomic status, and lifestyles.

## Conclusions

Collectively, results of the current meta-analysis reveal that EBV infection is associated with an increased risk of OSCC. Our study provides new insights in understanding OSCC pathogenesis and designing programs to prevent and treat OSCC. Further high-quality and larger sample studies are crucial to confirm the role of EBV in the pathogenesis of OSCC.

## Supporting information

S1 File PRISMA checklist. (DOC)

S1 Data Data for Stata. (XLSX)

## References

[1] Sinevici N, O'sullivan J. Oral cancer: Deregulated molecular events and their use as biomarkers. Oral Oncol. 2016; 61:12–8. http://dx.doi.org/10.1016/j.oraloncology. 2016.07.013. doi: 10.1016/j.oraloncology.2016.07.013 27688099 10.1016/j.oraloncology.2016.07.013

[2] Feller L, Lemmer J. Oral Squamous Cell Carcinoma: Epidemiology, Clinical Presentation and Treatment. J Cancer Ther. 2012; 03: 263–268. http://dx.doi.org/10.4236/jct.2012.34037.

[3] Zhong LP, Zhang CP, Ren GX, Guo W, William WN Jr, Hong CS, et al Long-term results of a randomized phase III trial of TPF induction chemotherapy followed by surgery and radiation in locally advanced oral squamous cell carcinoma. Oncotarget. 2015; 6: 18707–18714. http://dx.doi.org/10.18632/oncotarget.4531. doi: 10.18632/oncotarget.4531 26124084 10.18632/oncotarget.4531 PMC4621922

[4] Reichart PA, Nguyen XH. Betel quid chewing, oral cancer and other oral mucosal diseases in Vietnam: a review. J Oral Pathol Med. 2008; 37: 511–514. http://dx.doi.org/10.1111/j.1600-0714.2008.00669.x. doi: 10.1111/j.1600-0714.2008.00669.x 18624933 10.1111/j.1600-0714.2008.00669.x

[5] Marur S, Forastiere AA. Head and neck cancer: changing epidemiology, diagnosis, and treatment. Mayo Clin Proc. 2008; 83: 489–501. http://dx.doi.org/10.4065/83.4.489. doi: 10.4065/83.4.489 18380996 10.4065/83.4.489

[6] Goldstein BY, Chang SC, Hashibe M, Vecchia CL, Zhang ZF. Alcohol Consumption and Cancer of the Oral Cavity and Pharynx from 1988 to 2009: An Update. Eur J Cancer Prev. 2010; 19: 431 http://dx.doi.org/10.1097/CEJ.0b013e32833d936d. doi: 10.1097/CEJ.0b013e32833d936d 20679896 10.1097/CEJ.0b013e32833d936d PMC2954597

[7] Thompson MP, Kurzrock R. Epstein-Barr virus and cancer. Clin Cancer Res. 2004; 10: 803–821. http://dx.doi.org/10.1158/1078-0432.CCR-0670-3. 14871955 10.1158/1078-0432.ccr-0670-3

[8] Javier RT, Butel JS. The History of Tumor Virology. Cancer Research. 2008; 68: 7693 http://dx.doi.org/10.1158/0008-5472.CAN-08-3301. doi: 10.1158/0008-5472.CAN-08-3301 18829521 10.1158/0008-5472.CAN-08-3301 PMC3501656

[9] Prabhu SR, Wilson DF. Evidence of Epstein–Barr Virus Association with Head and Neck Cancers: A Review. J Can Dent Assoc. 2016; 82:g2 27548665

[10] Grywalska E, Rolinski J. Epstein-Barr Virus-Associated Lymphomas. Seminars in Oncology. 2015; 42: 291–303. https://doi.org/10.1053/j.seminoncol.2014.12.030. doi: 10.1053/j.seminoncol.2014.12.030 25843733 10.1053/j.seminoncol.2014.12.030

[11] Van Rensburg EJ, Engelbrecht S, Van HW, Raubenheimer E, Schoub BD. Detection of EBV DNA in oral squamous cell carcinomas in a black African population sample. In Vivo. 1995; 9: 199–202. 8562882

[12] van Heerden WE, van Rensburg EJ, Engelbrecht S, Raubenheimer EJ. Prevalence of EBV in oral squamous cell carcinomas in young patients. Anticancer Res. 1995; 15: 2335–2339. 8572648

[13] Cruz I, Rd VDBA, Snijders PJ, Meijer CJ, Walboomers JM, Snow GB, et al Prevalence of Epstein-Barr virus in oral squamous cell carcinomas, premalignant lesions and normal mucosa—a study using the polymerase chain reaction. Oral Oncol. 1997; 33: 182–188. http://dx.doi.org/10.1016/S0964-1955(96)00054-1. 9307727 10.1016/s0964-1955(96)00054-1

[14] Stroup DF, Berlin JA, Morton SC, Olkin I, Williamson GD, Rennie D, et al Meta-analysis of observational studies in epidemiology: a proposal for reporting. Meta-analysis Of Observational Studies in Epidemiology (MOOSE) group. JAMA. 2008; 283: 2008–2012. http://dx.doi.org/10.1001/jama.283.15.2008. 10789670 10.1001/jama.283.15.2008

[15] Wells GA, Shea BJ, O'Connell D, Peterson J, Welch V, Losos M, et al The Newcastle–Ottawa Scale (NOS) for Assessing the Quality of Non-Randomized Studies in Meta-Analysis. Available from: http://www.ohri.ca/programs/clinical_epidemiology/oxford.asp. Accessed 2 Apr 2017

[16] Higgins JPT, Thompson SG. Quantifying heterogeneity in a meta-analysis. Stat Med. 2002; 21: 1539–1558. http://dx.doi.org/10.1002/sim.1186. doi: 10.1002/sim.1186 12111919 10.1002/sim.1186

[17] Higgins JP, Thompson SG, Deeks JJ, Altman DG. Measuring inconsistency in meta-analyses. BMJ. 2003; 327(7414):557–560. http://10.1136/bmj.327.7414.557. doi: 10.1136/bmj.327.7414.557 12958120 10.1136/bmj.327.7414.557 PMC192859

[18] Dersimonian R, Laird N. Meta-analysis in clinical trials. Control Clin Trials. 1986; 7: 177–188. https://doi.org/10.1016/0197-2456(86)90046-2. 3802833 10.1016/0197-2456(86)90046-2

[19] Bagos PG, Nikolopoulos GK. Generalized least squares for assessing trends in cumulative meta-analysis with applications in genetic epidemiology. J Clin Epidemiol. 2009; 62: 1037–1044. http://dx.doi.org/10.1016/j.jclinepi.2008.12.008. doi: 10.1016/j.jclinepi.2008.12.008 19345563 10.1016/j.jclinepi.2008.12.008

[20] Begg CB, Mazumdar M. Operating characteristics of a rank correlation test for publication bias. Biometrics. 1994; 50: 1088–1101. http://dx.doi.org/10.2307/25334. 7786990

[21] Egger M, Smith GD, Schneider M, Minder C. Bias in meta-analysis detected by a simple, graphical test. BMJ. 1997; 315: 629–634. https://doi.org/10.1136/bmj.315.7109.629. 9310563 10.1136/bmj.315.7109.629 PMC2127453

[22] Sterne JA, Egger M. Funnel plots for detecting bias in meta-analysis: guidelines on choice of axis. J Clin Epidemiol. 2001; 54: 1046–1055. https://doi.org/10.1016/S0895-4356(01)00377-8 11576817 10.1016/s0895-4356(01)00377-8

[23] Ding XQ, Zhu ZY. Relationship between Epstein-Barr virus infection and oral squamous cell carcinoma. Acad J SUM S (in Chinese). 1997; 18: 140–141,153. http://dx.doi.org/10.13471/j.cnki.j.sun.yat-sen.univ(med.sci).1997.0046.

[24] Chen WL Tong LW, Deng QL. Human papillomavirus type 16 and Epstein-Barr virus relative to oral squamous cell carcinoma. J Oral Maxillofac Surg (in Chinese). 1998; 8: 23–25

[25] D'Costa J, Saranath D, Sanghvi V, Mehta AR. Epstein-Barr virus in tobacco-induced oral cancers and oral lesions in patients from India. J Oral Pathol Med. 1998; 27: 78–82. http://dx.doi.org/10.1111/j.1600-0714.1998.tb02098.x. 9526734 10.1111/j.1600-0714.1998.tb02098.x

[26] Shimakage M, Horii K, Tempaku A, Kakudo K, Shirasaka T, Sasagawa T. Association of Epstein-Barr virus with oral cancers. Hum Pathol. 2002; 33: 608–614. http://dx.doi.org/10.1053/hupa.2002.129786. 12152159 10.1053/hupa.2002.129786

[27] Sand LP, Jalouli J, Larsson PA, Hirsch JM. Prevalence of Epstein-Barr virus in oral squamous cell carcinoma, oral lichen planus, and normal oral mucosa. Oral Surg Oral Med Oral Pathol Oral Radiol Endod. 2002; 93: 586–592. http://dx.doi.org/10.1067/moe.2002.124462. 12075209 10.1067/moe.2002.124462

[28] Shamaa AA, Zyada MM, Wagner M, Awad SS, Osman MM, Abdel Azeem AA. The significance of Epstein Barr virus (EBV) & DNA topoisomerase II alpha (DNA-Topo II alpha) immunoreactivity in normal oral mucosa, oral epithelial dysplasia (OED) and oral squamous cell carcinoma (OSCC). Diagn Pathol. 2008; 3: 45 http://dx.doi.org/10.1186/1746-1596-3-45. doi: 10.1186/1746-1596-3-45 19021895 10.1186/1746-1596-3-45 PMC2611966

[29] Bagan JV, Jiménez Y, Murillo J, Poveda R, Díaz JM, Gavaldá C, et al Epstein-Barr virus in oral proliferative verrucous leukoplakia and squamous cell carcinoma: A preliminary study. Med Oral Patol Oral Cir Bucal. 2008; 13: E110–113. 18223526

[30] Kis A, Fehér E, Gáll T, Tar I, Boda R, Tóth ED, et al Epstein-Barr virus prevalence in oral squamous cell cancer and in potentially malignant oral disorders in an eastern Hungarian population. Eur J Oral Sci. 2009; 117: 536–540. http://dx.doi.org/10.1111/j.1600-0722.2009.00660.x. doi: 10.1111/j.1600-0722.2009.00660.x 19758249 10.1111/j.1600-0722.2009.00660.x

[31] Jiang R, Gu X, Moore-Medlin TN, Nathan CA, Hutt-Fletcher LM. Oral dysplasia and squamous cell carcinoma: Correlation between increased expression of CD21, Epstein-Barr virus and CK19. Oral Oncol. 2012; 48: 836–841. http://dx.doi.org/10.1016/j.oraloncology.2012.03.017. doi: 10.1016/j.oraloncology.2012.03.017 22513207 10.1016/j.oraloncology.2012.03.017 PMC3401344

[32] Kikuchi K, Noguchi Y, Hoshino M, Sakashita H, Yamada T, Inoue H, et al Detection of Epstein-Barr virus genome and latent infection gene expression in normal epithelia, epithelial dysplasia, and squamous cell carcinoma of the oral cavity. Tumour Biol. 2016; 37: 3389–3404. http://dx.doi.org/10.1007/s13277-015-4167-7. doi: 10.1007/s13277-015-4167-7 26449822 10.1007/s13277-015-4167-7

[33] Gupta K, Metgud R. Evidences suggesting involvement of viruses in oral squamous cell carcinoma. Patholog Res Int. 2013; 2013: 642496 http://dx.doi.org/10.1155/2013/642496. doi: 10.1155/2013/642496 24455418 10.1155/2013/642496 PMC3880768

[34] Mesri Enrique A, Feitelson MA, Munger K. Human Viral Oncogenesis: A Cancer Hallmarks Analysis. Cell Host Microbe. 2014; 15: 266–282. http://dx.doi.org/10.1016/j.chom.2014.02.011. doi: 10.1016/j.chom.2014.02.011 24629334 10.1016/j.chom.2014.02.011 PMC3992243

[35] Saravani S, Mirimoghaddam E, Sanadgol N, Kadeh H, Nazeri MR. Human Herpesvirus-6 and Epstein-Barr Virus Infections at Different Histopathological Grades of Oral Squamous Cell Carcinomas. Int J Prev Med. 2014; 5: 1231–1238. 25400880 PMC4223941

[36] Shimakage M, Sakamoto H. Macrophage involvement in Epstein-Barr virus-related tumors s. Exp Ther Med. 2010; 1: 285–291. http://dx.doi.org/10.3892/etm_00000044. doi: 10.3892/etm_00000044 22993541 10.3892/etm_00000044 PMC3445936

[37] Deacon EM, Matthews JB, Potts AJ, Hamburger J, Bevan IS, Young LS. Detection of Epstein-Barr virus antigens and DNA in major and minor salivary glands using immunocytochemistry and polymerase chain reaction: possible relationship with Sjogren's syndrome. J Pathol. 1991; 163: 351–360. http://dx.doi.org/10.1002/path.1711630413. doi: 10.1002/path.1711630413 1851828 10.1002/path.1711630413

[38] Adeyemi BF, Olusanya AA, Lawoyin JO. Oral squamous cell carcinoma, socioeconomic status and history of exposure to alcohol and tobacco. J Natl Med Assoc. 2011; 103: 498–502. http://dx.doi.org/10.1016/S0027-9684(15)30364-3. 21830633 10.1016/s0027-9684(15)30364-3

[39] Suresh K, Chandrashekara S. Sample size estimation and power analysis for clinical research studies. J Hum Reprod Sci. 2012; 5: 7–13. http://dx.doi.org/10.4103/0974-1208.97779. doi: 10.4103/0974-1208.97779 22870008 10.4103/0974-1208.97779 PMC3409926

[40] Hermann RM, Füzesi L, Pradier O, Christiansen H, Schmidberger H. Presence of human papillomavirus-18 and Epstein-Barr virus in a squamous cell carcinoma of the tongue in a 20-year-old patient. Case report and review of the current literature. Cancer Radiother. 2004; 8: 262–265. http://dx.doi.org/10.1016/j.canrad.2004.06.003. doi: 10.1016/j.canrad.2004.06.003 15450520 10.1016/j.canrad.2004.06.003

[41] Verma M. Infection and Cancer: Bi-Directorial Interactions. In: Shurin MR, Thanavala Y, Ismail N, editors. Multiple Infections and Cancer: Etiology, Mechanisms and Implications in Cancer Control Springer International Publishing; 2015 pp. 133–150. http://dx.doi.org/10.1007/987-3-319-20669_8

[42] Adler-Storthz K, Ficarra G, Woods KV, Gaglioti D, Dipietro M, Shillitoe EJ. Prevalence of Epstein-Barr virus and human papillomavirus in oral mucosa of HIV-Infected patients. J Oral Pathol Med. 1992; 21: 164–170. http://dx.doi.org/10.1111/j.1600-0714.1992.tb00095.x. 1318379 10.1111/j.1600-0714.1992.tb00095.x

[43] Horiuchi K, Mishima K, Ichijima K, Sugimura M, Ishida T, Kirita T. Epstein-Barr virus in the proliferative diseases of squamous epithelium in the oral cavity. Oral Surg Oral Med Oral Pathol Oral Radiol Endod. 1995; 79: 57–63. http://dx.doi.org/10.1016/S1079-2104(05)80075-7. 7614163 10.1016/s1079-2104(05)80075-7

[44] Liyanage SS, Rahman B, Gao Z, Zheng Y, Ridda I, Moa A, et al Evidence for the aetiology of human papillomavirus in oesophageal squamous cell carcinoma in the Chinese population: a meta-analysis. BMJ Open. 2013: e003604 http://dx.doi.org/10.1136/bmjopen-2013-003604. doi: 10.1136/bmjopen-2013-003604 24240141 10.1136/bmjopen-2013-003604 PMC3831092

[45] Jiang H, Yin J, Fan Y, Liu J, Zhang Z, Liu L, et al Gender difference in advanced HIV disease and late presentation according to European consensus definitions. Sci Rep, 2015; 5:14543 http://10.1038/srep14543. doi: 10.1038/srep14543 26412578 10.1038/srep14543 PMC4585954

[46] Nguyen SP, Bent S, Chen YH, Terdiman JP. Gender as a risk factor for advanced neoplasia and colorectal cancer: a systematic review and meta-analysis. Clin Gastroenterol Hepatol. 2009; 7(6):676–81.e1-e3. http://10.1016/j.cgh.2009.01.008. doi: 10.1016/j.cgh.2009.01.008 . 19514116 10.1016/j.cgh.2009.01.008

[47] Pham B, Klassen TP, Lawson ML, Moher D. Language of publication restrictions in systematic reviews gave different results depending on whether the intervention was conventional or complementary. J Clin Epidemiol, 2005; 58(8):769–776. http://dx.doi.org/10.1016/j.jclinepi.2004.08.021. 16086467 10.1016/j.jclinepi.2004.08.021

[48] Morrison A, Polisena J, Husereau D, Moulton K, Clark M, Fiander M, et al The effect of English-language restriction on systematic review-based meta-analyses: a systematic review of empirical studies. Int J Technol Assess Health Care. 2012; 28(2):138 http://10.1017/S0266462312000086. doi: 10.1017/S0266462312000086 22559755 10.1017/S0266462312000086

[49] Shi X, Zhang TT, Hu WP, Ji QH. Marital status and survival of patients with oral cavity squamous cell carcinoma: a population-based study. 2017; 8: 28526–28543. http://dx.doi.org/10.18632/oncotarget.16095. doi: 10.18632/oncotarget.16095 . 28415710 10.18632/oncotarget.16095 PMC5438670

