# Correction: Epstein-Barr virus infection and oral squamous cell carcinoma risk: A meta-analysis

**DOI:** 10.1371/journal.pone.0217659

**Published:** 2019-06-20

**Authors:** Yangyang She, Xiaolin Nong, Min Zhang, Menglin Wang

The images for Figs 3 and 5 are incorrectly switched. The image that appears as Fig 3 should be Fig 5, and the image that appears as Fig 5 should be Fig 3. The figure captions appear in the correct order.

**Fig 3:**
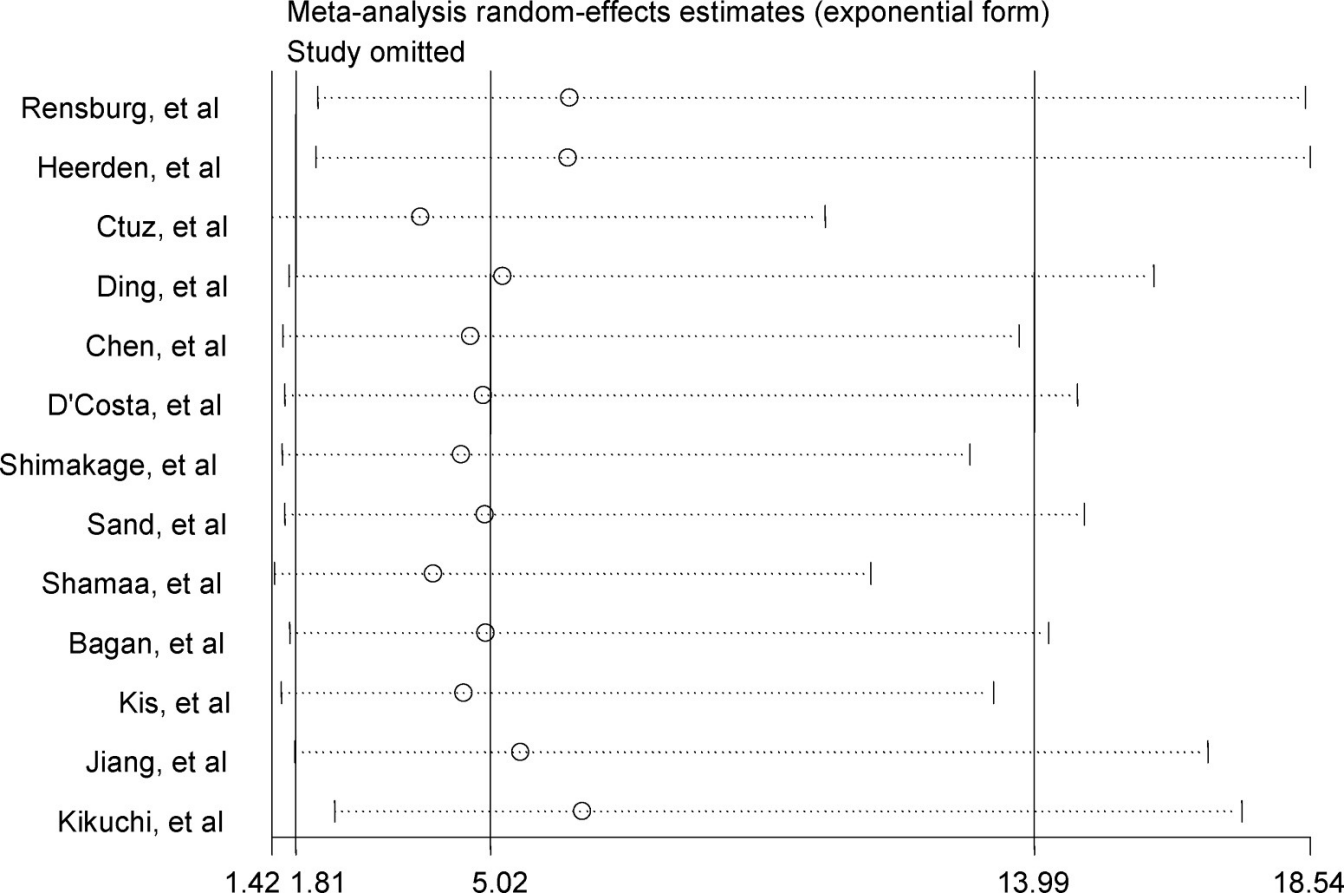
Sensitivity analyses by omitting individual study.

**Fig 5:**
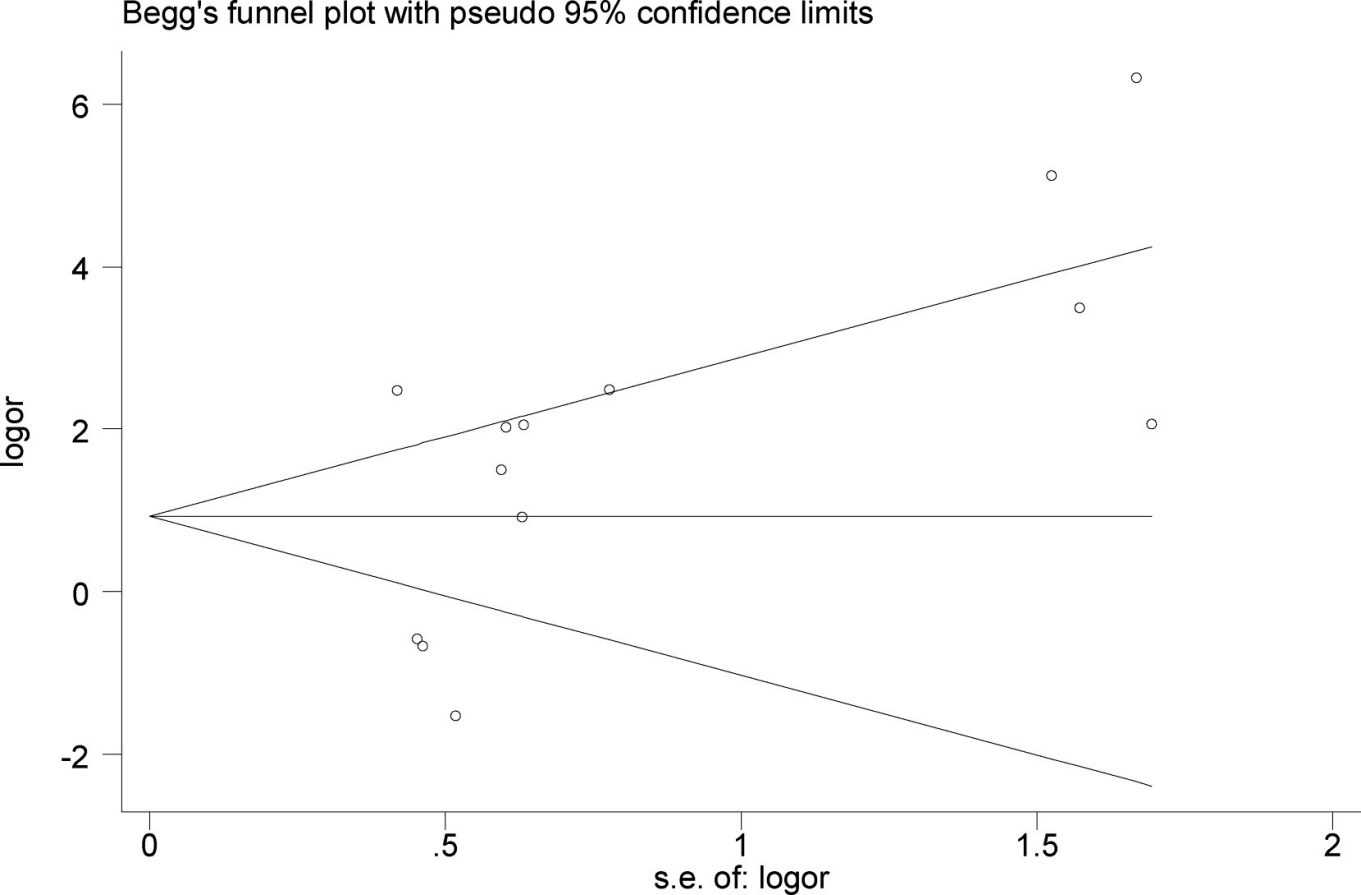
Funnel plot for publication bias regarding the association between EBV infection and OSCC risk.
